# Title: the impact of a pilot integrated care model on the quality and costs of inpatient care among chinese elderly: a difference-in-difference analysis of repeated cross-sectional data

**DOI:** 10.1186/s12962-022-00361-4

**Published:** 2022-06-25

**Authors:** Zhaojia Ye, Yawen Jiang

**Affiliations:** 1 Shenzhen Center for Disease Control and Prevention, Shenzhen, Guangdong China; 2 School of Public Health (Shenzhen), Sun Yat-sen University, Shenzhen, Guangdong China; 3 Sun Yat-sen University, 66 Gongchang Road, Guangming District, Shenzhen, Guangdong China

**Keywords:** Integrated care, China, Health maintenance organization, Elderly, Value

## Abstract

**Background:**

Recently, integrated care has received tremendous popularity in China, a leading example of which is the Luohu model. In the present analysis, we aimed to examine the impacts of the Luohu model on the quality and costs of inpatient care.

**Methods:**

We conducted a retrospective analysis using administrative claims databases of Shenzhen City (the city that the Luohu district sits) from Jan 2015–Apr 2017, which encompassed the time before and after the implementation of the pilot model. The outcomes were 30-day readmission, inpatient costs, and length of stay (LOS). Multivariable difference-in-difference analyses were conducted.

**Results:**

In the first year following the integration, the Luohu model did not have impacts on any of the outcomes. Although its effect on readmission (ratio of odds ratio: 1.082; 95% CI: 0.865 to 1.353) was still not identified in the first four months of the second post-integration year, it decreased inpatient costs by CN¥ 1224.1 (95% CI: 372.7 to 2075.5) and LOS by 0.938 days (95% CI: 0.0416 to 1.835) per hospitalization episode during the same period.

**Conclusions:**

The Luohu model may reduce costs and LOS in the long term. It is potentially a viable approach to improve the value of inpatient care in China.

**Supplementary Information:**

The online version contains supplementary material available at 10.1186/s12962-022-00361-4.

## Introduction

As with many middle-income countries, the healthcare system of China is seeing growing tension driven by aging population and changes in the profile of disease prevalence [1]. Characterized by inefficiency and fragmentation, the conventional healthcare systems in China were deemed suboptimal to meet the ever-increasing healthcare demand of the population with the currently limited resources [1]. To reinvigorate the healthcare systems under the new norms of population distribution and economic growth, the Chinese government institutionalized policies to encourage innovative healthcare models that can incentivize improved value of care. Specifically, the government introduced novelties that echoed patient-centered integrated care proposed by the World Health Organization [2]. At its core, patient-centered integrated care emphasizes the efficiency and quality of healthcare services by coordinating different sectors of the healthcare systems to steer away from the diagnosis-centered reactive care approach [3].

**Fig. 1:**
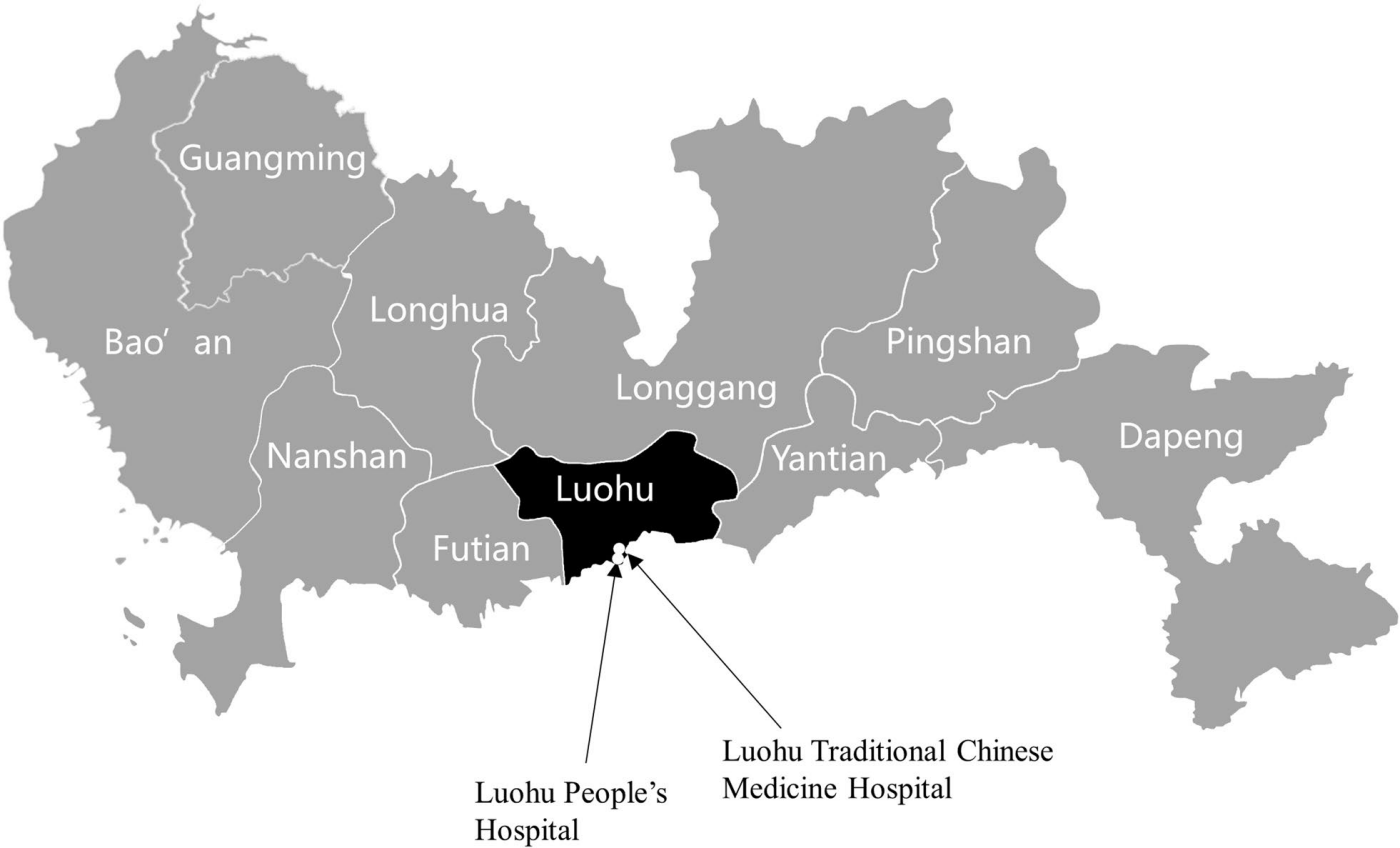
An overview of the districts in Shenzhen, China and the location of Luohu district. Luohu is one of the 10 districts in Shenzhen. Two comprehensive hospitals and several non-hospital facilities were integrated into the Luohu Hospital Group

Among the numerous pilot efforts, the most highlighted pioneering attempt was the Luohu Model, which was an analogue to the health maintenance organization (HMO) model in several aspects. HMOs aim to improve efficiency by committing to controlling costs and ensuring quality through coordinated management of patients [4]. The Luohu model attempted to include such features into its own design. Therefore, it was anecdotally referred to as the Luohu HMO model [5]. To provide some context, Luohu is a district (sub-city jurisdiction) in Shenzhen, a megacity in Southern China with over 12 million permanent residents [6]. While the overall population of Shenzhen is relatively young with only 6.5% of its residents aged over 60 years, Luohu hosts the largest elderly population among all districts [7, 8]. The Luohu model received its name from the Luohu Hospital Group (LHG), which was established in Aug 2015 by integrating five district-run hospitals and 23 community health centers in Luohu District of Shenzhen City [9]. Therefore, it was a hybrid model of vertical and horizontal integration. Among the five hospitals, two (Luohu People’s Hospital and Luohu Traditional Chinese Medicine Hospital) were comprehensive hospitals, one was a women and children’s health center, and the remaining two were facilities specializing in public health (Fig. 1). City-run hospitals located in Luohu were not subject to the integration. Like HMOs, LHG received bundled per-capita payment of the contracted district residents from the payer, the municipal medical security administration of Shenzhen City [5]. It also shared the principle of making profits by preventing medical conditions and occurrences [5]. However, it also set itself apart from HMOs with several sharp contrasts. First, LHG was prohibited by the government from medical underwriting such that people who were registered residents of the Luohu district and covered by social health insurance (SHI) in Shenzhen city were equally eligible regardless of pre-conditions [5]. Second, it put no restrictions on out-of-network care such that the expenditures of members incurred outside of the group were also reimbursable [5]. Taken together, the Luohu model was conceptually appealing by creating strong incentives of cost containment and quality improvement. As such, this model became popular among policymakers and scholars nationwide, following which it was copied to several other districts in Shenzhen since 2018 and featured in prestigious scholarly outputs [2, 10].

However, previous publications mostly focused on narrative introduction and qualitative assessment of the Luohu model. To date, empirical evidence on the quality and cost impacts of the Luohu model using natural experiments is still at large, which poses as important barriers to the understanding of the value of integrated care in China and the development of the healthcare systems towards value-based care. Showcased for hundreds of new hospital groups that are currently being established, the impacts of the Luohu model on the efficiency of care should be evaluated. As such, the present study aimed to insight into the effects of the Luohu model on the quality and costs of inpatient care among the elderly, the results of which will provide implications for the ongoing nationwide reforms in China that largely replicate the Luohu model. More, low- and middle-income countries (LMICs) commonly employ local rather than national coordination when integrating health services, which has been identified as one of the main limits to programs’ efficacy [11]. Evidence from the present study will also help to shed light on this issue, thereby benefiting the implementation of integrated care in other LMICs. We hypothesized that the Luohu model improved quality and reduced costs compared with the conventional practice.

## Materials and methods

### Data and study time frame

The study primarily used inpatient electronic administrative databases from Jan 1, 2015 to Apr 1, 2017 of persons at least 60 years old covered by SHI in Shenzhen, China, with the corresponding outpatient database engaged to provide baseline clinical information.

**Fig. 2:**
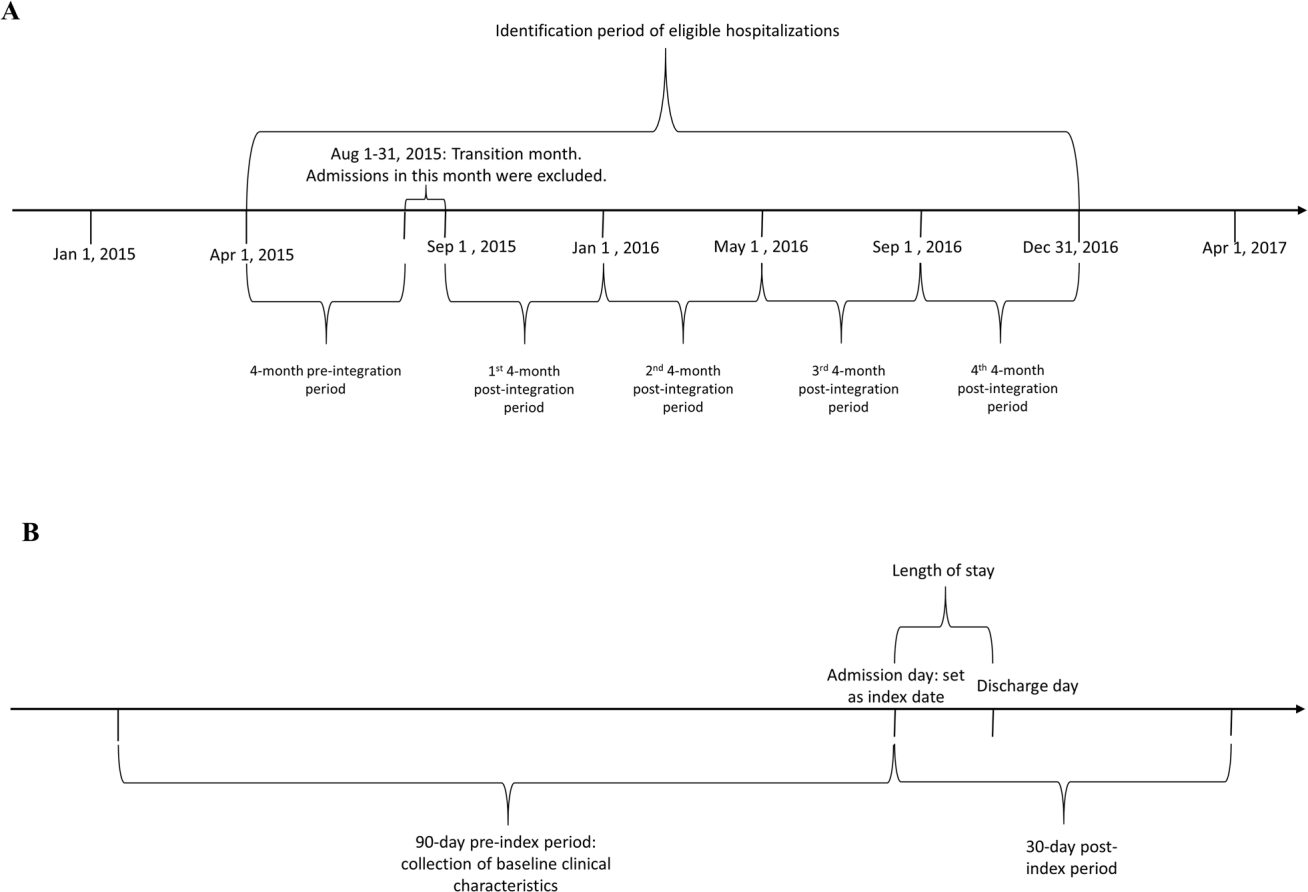
Design of study periods and the analytic periods of each observation. Panel A illustrates the definition of study periods and panel B depicts the time frame of each observation

To be eligible for the analysis, the admissions should have at least one properly formatted International Classification of Diseases 10th Revision (ICD-10) diagnosis code. Also, the admission day was set as the index date. In the present analysis, a 90-day pre-index period was used. Depending on the outcome under investigation, the post-index period could either be the 30-day period after the initial admission or the length of stay in the current study. To allow sufficient pre-index and post-index time periods, only admissions between Apr 1, 2015 and Dec 31, 2016 were included. More, the month of integration into LHG, Aug of 2015, was considered the transition month and excluded from the analysis. In relation to the transition month, the 4-month period of Apr–Jul 2015 was considered the pre-integration period, whereas Sep 2015–Dec 2016 was defined as the post-integration period and was split into four 4-month periods to allow the estimation of time-varying Luohu model effects in subsequent econometric analyses. The definition of study periods and the time frame of each observation are depicted in panels A and B of Fig. 2, respectively.

### Intervention and counterfactual groups

The intervention in the present study was the formation of LHG, which is referred to as integration hereinafter. Although LHG contained five hospitals, only the two comprehensive hospitals routinely admitted patients of all ages. The women and children’s health center specialized in maternal and pediatric services, while the public health-focused facilities did not admit inpatients. This was also confirmed by our data in that hospitalizations in the non-comprehensive hospitals were not identified. Accordingly, the intervention group was defined as admission to any of the two LHG-covered comprehensive hospitals and denoted by an indicator of LHG regardless of whether the integration was already implemented. Once the intervention group was identified, the rest of the observations were pooled as the comparison group.

### Outcomes

The outcomes in the present study were chosen to reflect quality and resource utilization of inpatient care. The attribute of quality was proxied by an indicator of 30-day readmission, which was a commonly used metric for this purpose [12, 13]. Also, the attribute of resource utilization was primarily represented by the total costs of the hospitalization. In addition, the length of stay was included as a surrogate endpoint of healthcare resource utilization because hospitalizations in China have persistently been plagued of prolonged low-value stays [14–16]. These outcomes have also been used as effectiveness measures of integrated care in other studies based on the postulation that improved coordination was supposed to reduce avoidable hospitalization and save costs [17, 18].Whereas Luohu model should presumably reduce readmission, costs, and length of stay, its value could also be entailed in reducing readmission while maintaining costs and length of stay or lowering costs and length of stay without sacrificing readmission rates.

Aside from the three outcomes of primary interest in the analysis of inpatient care, we also conducted an exploratory analysis of 90-day post-index total outpatient costs. To the extent that LHG was capable of shifting costs from inpatient setting to outpatient setting within its own organization, increased outpatient costs would raise concerns of the efficiency of the Luohu model even if inpatient costs decreased. Therefore, the additional analysis of outpatient care was conducted to examine whether there were crowded-out utilization from inpatient settings to the outpatient setting.

### Baseline characteristics and covariates

The baseline characteristics used in the analysis were age, sex, and several types of 90-day pre-index information including any hospitalization, days of inpatient stays, inpatient costs, number of outpatient visits, and outpatient costs. In addition, two sets of other covariates were used in the description and the multivariable analyses of index hospitalizations. The first set was indicators for each of the district in which the hospitalization occurred. In the multivariable analyses, the district of Luohu was the reference category. The second set contained 15 indicators of whether specific diagnoses were documented during the index hospitalization. It is noteworthy that almost 90% of the records had more than one diagnoses, none of which could be distinguished as the primary diagnosis. Therefore, we did not account for primary diagnoses in the analysis. When creating covariates based on diagnoses, it was also necessary to limit the list of conditions to a manageable scope. To determine the types of diagnoses to take into account, we tabulated the category (first three characters) of ICD-10 codes and selected the most prevalent 15 categories, which are listed in Additional file 1: Table S1. All categories that were not among the 15 most prevalent ones were collectively denoted by an indicator of “all other diagnoses”, which was used as the reference category in multivariable analyses.

### Empirical strategies

To describe discrete and continuous variables including both characteristics and outcomes, percentages and mean (SD) were calculated, respectively. Also, t-tests and ${\chi }^{2}$-tests were conducted to test the differences in baseline characteristics and outcomes across the intervention and comparison groups.

The main purpose of the empirical analysis was to identify causal impacts of Luohu model on the three outcomes. Since the assignment of individuals to the intervention and comparison groups was not random, it was important to minimize potential omitted variable bias. To that end, we used difference-in-difference (diff-in-diff) frameworks on top of multivariable regressions that controlled for all the observed baseline and clinical characteristics. The diff-in-diff frameworks enabled researchers to identify treatment effects by accounting for time-invariant unobserved endogenous variables as well as common time trends across groups [19–21]. Specifically, each of the four 4-month post-integration periods were interacted with the LGH indicator. The first three periods comprised the first post-integration year, during which the short-term effects were analyzed. The fourth period, on the other hand, represented effects in a relatively long term. As such, the diff-in-diff specification took the following form

$$E\left( Y \right) = g\left( {\alpha + \sum\limits_{t = 1}^T {{\beta _t}} pos{t_t} + \rho LHG + \sum\limits_{t = 1}^T {{\delta _t}} \times pos{t_t} \times LHG + X'\gamma } \right),$$

where $Y$ was a specific outcome, $LHG$ was an indicator for the intervention group, $pos{t}_{t}$ represented indicators for the four post-integration periods in which t took an integer value between 1 and 4, $X$ was a vector of covariates, ${\beta }_{t}$ was the post-integration time period fixed effects, $\rho$was the fixed effect of the LHG hospitals, ${\delta }_{t}$ were the Luohu model effects in the post-integration periods that were of primary interest in the present study, $\gamma$ was the coefficients of the covariates, and $g$ was the functional form that links the outcome and the linear sum of the covariates.

To implement the analytic framework specified above, we first conducted ordinary least square (OLS) regressions using robust standard errors. The coefficients of the interaction terms from OLS estimation could directly be interpreted as the Luohu model effects without the need of parameter transformation.

However, OLS estimates might be inefficient and sensitive when analyzing skewed outcomes such as costs and length of stay [22]. Therefore, we used alternative regression techniques that were relatively efficient and robust compared with OLS [22]. Specifically, we conducted a logistic regression for 30-day readmission, a generalized linear model (GLM) with gamma distribution and log link for inpatient costs, and a negative binomial regression for length of stay, all of which engaged the same diff-in-diff specification [22, 23]. In the logistic regression, the effect of Luohu model was represented by the exponential of each interaction term, which should be interpreted as the ratio of odds ratios (ROR). Intuitively, it describes how the time effect on 30-day readmission in relation to the pre-integration period in the intervention group compared to that in the comparison group. If the 30-day readmission rate dropped faster or increased slower in the intervention group, then ROR would be less than 1.

In the GLM and negative binomial regressions, the marginal effects or incremental effects of the explanatory variables on the outcomes could be estimated by taking the partial derivative or its discrete analog if only main effects were of interest. However, such metrics could not be obtained for an interaction term the same way as that of first-order variables [24]. As such, the discrete interaction effects, which were the differences between the time period effects conditional on the individuals being in the Luohu model and those conditional on the individuals not being in the Luohu model, were calculated [24]. These quantities represented the time-varying Luohu model effects during the pre-specified post-integration time periods.

The assumptions of parallel pre-intervention time trends were visual inspected and statistically tested by conducting regressions using month by LHG indicator interaction terms using the restricted samples of pre-integration period.

## Results

Out of 158,251 hospitalizations of SHI-covered individuals who aged 60 years and older at any point between Jan 1, 2015 and Apr 1, 2017, 120,081 hospitalizations met the inclusion and exclusion criteria. Among these, 5,701 (4.75%) were in the Luohu model intervention group. The flowchart of sample selection is shown in Additional file 1: Fig. S1.

The descriptive statistics and tests of baseline characteristics are presented in Table 1. The two groups had statistically significant differences in the percentages of male patients (47.43% vs. 49.66%, p = 0.001), the proportions that had any 90-day pre-index hospitalizations (25.03% vs. 31.13%, p < 0.001), and 90-day average pre-index inpatient costs [CN¥ 6166.1 (SD: 18596.8) vs. 7459.4 (SD: 20628.0), p < 0.001]. In addition, the percentage of hospitalizations that were associated with each of 11 out of 15 types of diagnoses was significantly different across the two groups. To an extent, such unbalances raised concerns that systematic cross-group heterogeneity that were not observed in the data could not be ruled out.

**Table 1:** Baseline characteristics of hospitalized patients within and outside of Luohu HMO and descriptive statistics of outcomes

	Luohu HMO (N = 5701)	Not Luohu HMO (N = 115,100)	Total (N = 120,801)	p-value
Age (years)	71.87 (8.73)	72.01 (8.68)	72.01 (8.68)	0.235
Male (%)	47.43	49.66	49.56	0.001
Any hospitalization in the 90-day pre-index period (%)	25.03	31.13	30.84	< 0.001
Days of inpatient stays in the 90-day pre-index period	5.31 (13.36)	5.59 (12.39)	5.57 (12.44)	0.106
Inpatient costs in the 90-day pre-index period (CN¥)	6166.10 (18596.75)	7459.41 (20628.04)	7398.38 (20538.46)	< 0.001
Number of outpatient visits in the 90-day pre-index period	2.05 (3.92)	2.12 (3.93)	2.12 (3.93)	0.187
Outpatient costs in the 90-day pre-index period (CN¥)	912.62 (2465.51)	976.79 (4565.90)	973.76 (4488.94)	0.292
Indicators of diagnoses during the index hospitalization (%)				
* Essential hypertension*	60.09	51.27	51.69	< 0.001
Type 2 diabetes	29.63	24.33	24.58	< 0.001
* Chronic ischemic heart disease*	18.98	21.25	21.14	< 0.001
Atherosclerosis	11.38	17.06	16.79	< 0.001
Disorders of lipoprotein metabolism and other lipidemias	27.31	15.72	16.27	< 0.001
Other liver diseases *(non-alcoholic fatty liver, congestion of liver, infarction of liver, etc.)*	14.80	14.55	14.56	0.599
Heart failure	9.72	13.19	13.03	< 0.001
Cerebral infarction	6.88	11.01	10.82	< 0.001
Sequelae of cerebrovascular disease	19.33	9.93	10.38	< 0.001
Other disorders of fluid, electrolyte and acid-base balance (hyperosmolality and hypernatremia, acidosis, alkalosis, etc.)	10.93	7.71	7.87	< 0.001
Gastritis and duodenitis	8.82	9.41	9.38	0.139
Other cerebrovascular diseases (other cerebrovascular diseases, other cerebrovascular diseases, etc.)	9.91	8.24	8.32	< 0.001
Cholelithiasis	7.95	8.03	8.03	0.810
Other disorders of kidney and ureter (ischemia and infarction of kidney, cyst of kidney, etc.)	9.51	9.33	9.34	0.648
Encounter for other aftercare and medical care	3.86	8.27	8.07	< 0.001
Outcomes				
30-day readmission (%)	12.59	16.65	16.46	< 0.001
Inpatient costs (CN¥)	14855.83 (29304.74)	16349.73 (32233.39)	16279.23 (32102.64)	< 0.001
Length of stay (days)	13.37 (14.95)	12.36 (14.71)	12.41 (14.72)	< 0.001

*HMO* health maintenance organization

Based on the descriptive statistics of the outcomes in Table 1, LHG hospitalizations had a lower 30-day readmission rate (12.59% vs. 16.65%, p < 0.001), lower inpatient costs [CN¥ 14855.8 (SD: 29304.7) vs. 16349.7 (SD: 32233.4), p < 0.001], and a longer average stay [13.4 (SD: 14.9) vs. 12.4 (SD: 14.7) days, p < 0.001].

The results of multivariable diff-in-diff analyses using OLS are displayed in Table 2 (full results with estimates of all covariates presented in Additional file 1: Table S2), the top section of which presented the interaction effects of interest. None of the three 4-month time periods within the first year had significant interaction effects with the LHG indicator on any of the outcomes, suggesting an absence of evidence on the Luohu model effects in the short term. However, the Luohu model did reduce inpatient costs by CN¥ 2032.8 (95% CI: 152.8 to 3912.9) and the length of stay by 1.356 days (95% CI: 0.501 to 2.211) in the fourth post-integration period. During the same period, its impact on the probability of 30-day readmission was still insignificant (0.00776, 95% CI: −0.0155 to 0.0310).

**Table 2:** Results of OLS regressions using the diff-in-diff specification

	30-day readmission	Hospitalization costs	Length of stay
Luohu HMO х1st post-integration period	− 0.00803 [− 0.0337, 0.0177]	477.9 [− 2150.3, 3106.1]	0.279 [− 1.232, 1.789]
Luohu HMO х2nd post-integration period	0.00395 [− 0.0190, 0.0269]	− 291.5 [− 2365.6, 1782.7]	0.294 [− 0.809, 1.397]
Luohu HMO х3rd post-integration period	0.0172 [− 0.00771, 0.0421]	90.34 [− 2410.6, 2591.3]	− 0.693 [− 2.012, 0.627]
Luohu HMO х4th post-integration period	0.00776 [− 0.0155, 0.0310]	− 2032.8^*^ [− 3912.9, − 152.8]	− 1.356^**^ [− 2.211, − 0.501]
1st post-integration period	− 0.00666^*^ [− 0.0130, − 0.000300]	446.0 [− 198.2, 1090.1]	− 0.0524 [− 0.342, 0.237]
2nd post-integration period	− 0.00665^*^ [− 0.0121, − 0.00117]	86.49 [− 409.3, 582.3]	− 0.486^***^ [− 0.715, − 0.257]
3rd post-integration period	− 0.0126^***^ [− 0.0188, − 0.00647]	651.8^*^ [79.79,1223.9]	− 0.0298 [− 0.296, 0.236]
4th post-integration period	− 0.00721^*^ [− 0.0128, − 0.00167]	441.3 [− 17.52, 900.2]	− 0.390^***^ [− 0.589, − 0.191]
Luohu HMO Indicator	− 0.0115 [− 0.0296,0.00659]	− 5302.3^***^ [− 7089.4,-3515.1]	0.548 [− 0.553,1.649]
*N*	120,801	120,801	120,801

Standard errors in parentheses

^*^ *p* < 0.05, ^**^ *p* < 0.01, ^***^ *p* < 0.001

*OLS* ordinary least squares; *HMO* health maintenance organization

The estimates of Luohu model effects using logistic, GLM, and negative binomial regressions with the diff-in-diff specification are listed in Table 3 (full results with estimates of all covariates presented in Additional file 1: Table S3). Similar to the OLS outputs, the Luohu model did not have impacts on any of the outcomes during the first year but decreased inpatient costs by CN¥ 1224.1 (95% CI: 372.7 to 2075.5) and the length of stay by 0.938 days (95% CI: 0.0416 to 1.835) in the fourth post-integration period. By contrast, the ROR of the fourth post-integration period by the LHG indicator interaction term was insignificant at 1.082 (95% CI: 0.865 to 1.353).

**Table 3:** Logistic and generalized linear model regression results using the diff-in-diff specification

	30-day readmission	Costs of hospitalization episode [marginal effects/incremental effects (CI)]	Length of stay [marginal effects/incremental effects (CI)]
Luohu HMO х1st post-integration period [ROR (CI)]	0.895 [0.679, 1.181]	518.2 [− 573.0, 1609.3]	0.346 [− 0.784, 1.475]
Luohu HMO х2nd post-integration period [ROR (CI)]	1.028 [0.821, 1.287]	341.3 [− 542.3, 1224.9]	0.662 [− 0.280, 1.604]
Luohu HMO х3rd post-integration period [ROR (CI)]	1.168 [0.915, 1.491]	657.3 [-376.5, 1691.0]	− 0.118 [− 1.157, 0.920]
Luohu HMO х4th post-integration period [ROR (CI)]	1.082 [0.865, 1.353]	− 1224.1^**^ [− 2075.5, − 372.7]	− 0.938^*^ [− 1.835, − 0.0416]
1st post-integration period	0.943^*^ [0.895, 0.993]	471.7^**^ [173.0, 770.4]	− 0.144 [− 0.373, 0.0854]
2nd post-integration period	0.944^*^ [0.902, 0.987]	130.4 [− 121.0, 381.8]	− 0.561^***^ [− 0.757, − 0.364]
3rd post-integration period	0.898^***^ [0.853, 0.945]	664.4^***^ [375.1, 953.7]	− 0.157 [− 0.378, 0.0638]
4th post-integration period	0.938^**^ [0.895, 0.983]	254.9 [− 4.165, 514.0]	− 0.478^***^ [− 0.677, − 0.280]
Luohu HMO Indicator	0.889 [0.744, 1.063]	− 4879.0^***^ [− 5259.4, − 4498.6]	0.206 [− 0.182, 0.594]
*N*	120,801	120,801	120,801

95% confidence intervals in brackets

^*^ *p* < 0.05, ^**^ *p* < 0.01, ^***^ *p* < 0.001

*OR* odds ratio, *CI* 95% confidence interval, *HMO* health maintenance organization, *ROR* ratio of odds ratio

The results of the exploratory analysis of outpatient costs are shown in Additional file 1: Table S4. In this set of analysis, Luohu model did not impact outpatient costs in the first year but decreased 90-day post-index outpatient costs in the fourth post-integration period (CN¥ −145.5, 95% CI: −6.734 to −284.2). More, the pre-index trends of the outcomes in the two groups are illustrated in Additional file 1: Table S5; Figs. S2–S4. In the regressions, none of the month by LHG indicator interaction terms was significant, suggesting an absence of statistical evidence of violating the parallel trend assumptions in the pre-intervention period. In the plots, the trends in the two groups had good similarity overall although not perfectly parallel, which provided additional credit to the parallel trend assumptions.

## Discussion

Internationally, integrated care has generally been associated with reduced costs or improved quality [25, 26]. Although integrated care is becoming increasingly popular in China, its value has not been evidenced empirically previously. In the present study, we documented the effects of a novel integrated care system on the value of inpatient care. Our findings suggested that the Luohu HMO model, which was a leading example of integrated care in China, did reduce inpatients costs and length of stay. Unlike our hypotheses, the evidence did not suggest that the Luohu model also improved quality of inpatient care. However, this should not be interpreted as that the Luohu model did not enhance the efficiency of care. The evidence taken together, the Luohu model saved costs and constrained healthcare resource utilization without sacrificing quality. These findings suggest that the ongoing reforms replicating the Luohu approach elsewhere in China may help to curb the inpatient care expenditure. To our knowledge, the present study represented the first attempt to provide quantitative empirical evidence on the value of integrated care in China.

We did not find evidence that the Luohu model effect was onset in the first year after policy implementation. Indeed, the unlikelihood of finding evidence on effectiveness was spotted as one of the main challenges of analyzing the Luohu model in commentaries in the literature [27]. To that end, we also investigated the effects of the Luohu model in a relatively long term, during which the Luohu model started to manifest nontrivial impacts. It remains unknown whether greater effect sizes on quality of care could be captured had the post-integration period been extended in the study, which should be one of the research priorities in future agendas of this field. To examine whether the costs of care were simply squeezed to the outpatient care setting, we also analyzed outpatient costs using the same analytic frame. However, we did not identify such evidence.

Several limitations should be noted when interpreting the results. First, the types of outcomes to proxy the value of care were hardly exhaustive. Evidence generated using additional outcomes such as in-hospital and post-discharge mortality could impart greater validity to the results. Second, the number of institutions within the LHG was modest, which might limit the generalizability of the study. Third, the analysis could not delineate the effects of the Luohu model on specific types of service within the inpatient setting or investigate the sequential effects of the model from preventive care to disease management and inpatient care. A major consequence was that the rationale by which the model improved efficiency could not be ascertained. In a nutshell, several evidentiary gaps remain to be filled in future. Despite such limitations, the current findings carried important implications for healthcare policymaking in China.

## Conclusions

The pilot integrated care program of Luoho HMO model in Shenzhen, China reduced inpatient costs and length of hospital stay without sacrificing the decisive quality metrics such of 30-day readmission among the elderly population. Therefore, expanding the Luohu model to elsewhere in China may be a viable option to promote value-based healthcare.

## Supplementary Information

**Additional file 1: Table S1**. ICD-10 codes used in the identification of diagnoses associated with the index hospitalizations. **Table S2**. Full results of OLS regressions using the diff-in-diff specification. **Table S3**. Full results of the logistic and generalized linear model regression using the diff-in-diff specification. **Table S4**. Results of the OLS regression of 90-day post-index outpatient costs using the Diff-in-diff specification. **Table S5**. Results of the tests of parallel trends across groups using multivariate OLS regressions by including Luohu model indicator by month interaction terms. **Fig. S1**. The flowchart of sample selection. **Fig. S2**. The pre-index trends of 30-day readmission rates in the intervention and counterfactual groups. **Fig. S3**. The pre-index trends of inpatient costs among the intervention and counterfactual groups. **Fig. S4**. The pre-index trends of length of stays among the intervention and counterfactual groups.

## Data Availability

The datasets used or analyzed during the current study are available from the corresponding author on reasonable request.
