# Supplementary material for: Title: the impact of a pilot integrated care model on the quality and costs of inpatient care among chinese elderly: a difference-in-difference analysis of repeated cross-sectional data
Source: Cost Eff Resour Alloc. 2022 Jun 25;20:28. doi: 10.1186/s12962-022-00361-4 (PMC9233857; doi:10.1186/s12962-022-00361-4)
Supplement: Supplementary file 1 — Additional file 1: Table S1. ICD-10 codes used in the identification of diagnoses associated with the index hospitalizations. Table S2. Full results of OLS regressions using the diff-in-diff specification. Table S3. Full results of the logistic and generalized linear model regression using the diff-in-diff specification. Table S4. Results of the OLS regression of 90-day post-index outpatient costs using the Diff-in-diff specification. Table S5. Results of the tests of parallel trends across groups using multivariate OLS regressions by including Luohu model indicator by month interaction terms. Fig. S1. The flowchart of sample selection. Fig. S2. The pre-index trends of 30-day readmission rates in the intervention and counterfactual groups. Fig. S3. The pre-index trends of inpatient costs among the intervention and counterfactual groups. Fig. S4. The pre-index trends of length of stays among the intervention and counterfactual groups. [file 12962_2022_361_MOESM1_ESM.docx]

Table S1. ICD-10 codes used in the identification of diagnoses associated with the index hospitalizations

| Item NO. | condition | First 3 digits of code |
| --- | --- | --- |
| 1 | Essential hypertension | I10 |
| 2 | Type 2 diabetes | E11 |
| 3 | Chronic ischemic heart disease | I25 |
| 4 | Atherosclerosis | I70 |
| 5 | Disorders of lipoprotein metabolism and other lipidemias | E78 |
| 6 | Other liver diseases  (non-alcoholic fatty liver, congestion of liver, infarction of liver, etc.) | K76 |
| 7 | Heart failure | I50 |
| 8 | Cerebral infarction | I63 |
| 9 | Sequelae of cerebrovascular disease | I69 |
| 10 | Other disorders of fluid, electrolyte and acid-base balance (hyperosmolality and hypernatremia, acidosis, alkalosis, etc.) | E87 |
| 11 | Gastritis and duodenitis | K29 |
| 12 | Other cerebrovascular diseases (other cerebrovascular diseases, other cerebrovascular diseases, etc.) | I67 |
| 13 | Cholelithiasis | K80 |
| 14 | Other disorders of kidney and ureter (ischemia and infarction of kidney, cyst of kidney, etc.) | N28 |
| 15 | Encounter for other aftercare and medical care | Z51 |

Table S2. Full results of OLS regressions using the diff-in-diff specification

|  | 30-day readmission | Hospitalization costs | Length of stay |
| --- | --- | --- | --- |
| Luohu HMO х1^st^ post-integration period | -0·00803  [-0·0337,0·0177] | 477·9  [-2150·3,3106·1] | 0·279  [-1·232,1·789] |
| Luohu HMO х2^nd^ post-integration period | 0·00395  [-0·0190,0·0269] | -291·5  [-2365·6,1782·7] | 0·294  [-0·809,1·397] |
| Luohu HMO х3^rd^ post-integration period | 0·0172  [-0·00771,0·0421] | 90·34  [-2410·6,2591·3] | -0·693  [-2·012,0·627] |
| Luohu HMO х4^th^ post-integration period | 0·00776  [-0·0155,0·0310] | -2032·8^*^  [-3912·9,-152·8] | -1·356^**^  [-2·211,-0·501] |
| 1^st^ post-integration period | -0·00666^*^  [-0·0130,-0·000300] | 446·0  [-198·2,1090·1] | -0·0524  [-0·342,0·237] |
| 2^nd^ post-integration period | -0·00665^*^  [-0·0121,-0·00117] | 86·49  [-409·3,582·3] | -0·486^***^  [-0·715,-0·257] |
| 3^rd^ post-integration period | -0·0126^***^  [-0·0188,-0·00647] | 651·8^*^  [79·79,1223·9] | -0·0298  [-0·296,0·236] |
| 4^th^ post-integration period | -0·00721^*^  [-0·0128,-0·00167] | 441·3  [-17·52,900·2] | -0·390^***^  [-0·589,-0·191] |
| Luohu HMO Indicator | -0·0115  [-0·0296,0·00659] | -5302·3^***^  [-7089·4,-3515·1] | 0·548  [-0·553,1·649] |
| Age (years) | 0·000819^***^  [0·000571,0·00107] | 97·23^***^  [72·77,121·7] | 0·0430^***^  [0·0315,0·0544] |
| Male | 0·0126^***^  [0·00868,0·0166] | 1990·4^***^  [1633·3,2347·6] | 0·512^***^  [0·352,0·672] |
| Any hospitalization in the 90-day pre-index period | 0·0491^***^  [0·0424,0·0558] | 1788·4^***^  [1077·0,2499·7] | -0·0329  [-0·354,0·288] |
| Days of inpatient stays in the 90-day pre-index period | 0·00398^***^  [0·00362,0·00435] | -153·3^***^  [-212·7,-93·97] | 0·164^***^  [0·140,0·188] |
| Inpatient costs in the 90-day pre-index period (1,000 CN¥) | 0·000455^***^  [0·000253,0·000657] | 182·7^***^  [121·6,243·8] | -0·00330  [-0·0243,0·0177] |
| Number of outpatient visits in the 90-day pre-index period | 0·00200^***^  [0·00139,0·00261] | -171·6^**^  [-282·9,-60·43] | -0·105^***^  [-0·132,-0·0789] |
| Outpatient costs in the 90-day pre-index period (1,000 CN¥) | -0·000147  [-0·000542,0·000247] | 177·1^*^  [3·128,351·0] | 0·0314^**^  [0·00888,0·0540] |
| Indicators of diagnoses during the index hospitalization |  |  |  |
| *Essential hypertension* | 0·00833^***^  [0·00414,0·0125] | -198·6  [-596·4,199·2] | 0·220^*^  [0·0410,0·400] |
| *Type 2 diabetes* | 0·00495^*^  [0·000220,0·00969] | 1314·1^***^  [850·1,1778·2] | 0·997^***^  [0·792,1·202] |
| *Chronic ischemic heart disease* | -0·00126  [-0·00693,0·00441] | 266·5  [-319·0,852·0] | -0·293^*^  [-0·551,-0·0364] |
| *Atherosclerosis* | -0·0180^***^  [-0·0232,-0·0128] | -2457·7^***^  [-2902·5,-2012·8] | -0·177  [-0·402,0·0489] |
| *Disorders of lipoprotein metabolism and other lipidemias* | -0·0115^***^  [-0·0165,-0·00646] | -2171·8^***^  [-2582·2,-1761·3] | -0·707^***^  [-0·931,-0·483] |
| *Other liver diseases*  *(non-alcoholic fatty liver, congestion of liver, infarction of liver, etc.)* | -0·0285^***^  [-0·0338,-0·0232] | -1965·5^***^  [-2406·5,-1524·6] | -0·163  [-0·371,0·0453] |
| *Heart failure* | 0·00687  [-0·000154,0·0139] | 7481·5^***^  [6624·1,8338·9] | 0·138  [-0·191,0·466] |
| *Cerebral infarction* | 0·00891^**^  [0·00237,0·0155] | 3461·5^***^  [2756·6,4166·4] | 2·685^***^  [2·359,3·011] |
| *Sequelae of cerebrovascular disease* | 0·0271^***^  [0·0200,0·0342] | 5222·2^***^  [4407·5,6037·0] | 6·435^***^  [5·964,6·905] |
| *Other disorders of fluid, electrolyte and acid-base balance (hyperosmolality and hypernatremia, acidosis, alkalosis, etc.)* | 0·00870^*^  [0·000674,0·0167] | 7635·9^***^  [6746·4,8525·4] | 3·558^***^  [3·146,3·970] |
| *Gastritis and duodenitis* | -0·0251^***^  [-0·0311,-0·0192] | -3538·5^***^  [-3931·4,-3145·6] | -0·644^***^  [-0·835,-0·454] |
| *Other cerebrovascular diseases (other cerebrovascular diseases, other cerebrovascular diseases, etc.)* | -0·00945^**^  [-0·0161,-0·00278] | -3334·7^***^  [-3866·3,-2803·0] | -0·861^***^  [-1·145,-0·577] |
| *Cholelithiasis* | 0·000764  [-0·00652,0·00805] | 1097·2^**^  [426·0,1768·5] | 0·774^***^  [0·459,1·088] |
| *Other disorders of kidney and ureter (ischemia and infarction of kidney, cyst of kidney, etc.)* | 0·000432  [-0·00644,0·00730] | -418·7  [-1014·9,177·4] | 0·460^**^  [0·162,0·758] |
| *Encounter for other aftercare and medical care* | 0·328^***^  [0·317,0·338] | -5586·2^***^  [-6494·5,-4677·9] | -3·782^***^  [-4·152,-3·413] |
| District indicators |  |  |  |
| *Guangming* | 0·0270^***^  [0·0192,0·0348] | -5604·2^***^  [-6334·8,-4873·6] | 1·253^***^  [0·897,1·609] |
| *Nanshan* | 0·0869^***^  [0·0658,0·108] | -10938·2^***^  [-12176·6,-9699·7] | 4·818^***^  [3·425,6·212] |
| *Pingshan* | 0·0261^***^  [0·0206,0·0315] | -4102·1^***^  [-4671·8,-3532·5] | -1·278^***^  [-1·494,-1·061] |
| *Dapeng* | 0·0336^***^  [0·0187,0·0486] | -9316·3^***^  [-10385·1,-8247·4] | 0·0176  [-0·548,0·583] |
| *Bao’an* | 0·00786  [-0·000165,0·0159] | -4387·9^***^  [-5172·7,-3603·1] | 2·970^***^  [2·542,3·397] |
| *Yantian* | 0·00955  [-0·00501,0·0241] | -7889·6^***^  [-8950·1,-6829·2] | 0·226  [-0·388,0·840] |
| *Futian* | -0·00218  [-0·0100,0·00565] | -6244·2^***^  [-6936·5,-5551·8] | 0·156  [-0·175,0·486] |
| *Longhua* | 0·0349^***^  [0·0175,0·0522] | -11526·7^***^  [-12474·1,-10579·2] | -1·933^***^  [-2·561,-1·305] |
| *Longgang* | 0·0136  [-0·00410,0·0313] | -7533·0^***^  [-8845·9,-6220·0] | 1·687^***^  [1·030,2·344] |
| *N* | 120801 | 120801 | 120801 |

Standard errors in parentheses

^*^ *p* < 0·05, ^**^ *p* < 0·01, ^***^ *p* < 0·001

Abbreviations: OLS, ordinary least squares; HMO, health maintenance organization.

Table S3. Full results of the logistic and generalized linear model regression using the diff-in-diff specification.

|  | 30-day readmission | Costs of hospitalization episode [marginal effects/incremental effects (CI)] | Length of stay [marginal effects/incremental effects (CI)] |
| --- | --- | --- | --- |
| Luohu HMO х1^st^ post-integration period [ROR (CI)] | 0·895  [0·679,1·181] | 518·2  [-573·0,1609·3] | 0·346  [-0·784,1·475] |
| Luohu HMO х2^nd^ post-integration period [ROR (CI)] | 1·028  [0·821,1·287] | 341·3  [-542·3,1224·9] | 0·662  [-0·280,1·604] |
| Luohu HMO х3^rd^ post-integration period [ROR (CI)] | 1·168  [0·915,1·491] | 657·3  [-376·5,1691·0] | -0·118  [-1·157,0·920] |
| Luohu HMO х4^th^ post-integration period [ROR (CI)] | 1·082  [0·865,1·353] | -1224·1^**^  [-2075·5,-372·7] | -0·938^*^  [-1·835,-0·0416] |
| 1^st^ post-integration period | 0·943^*^  [0·895,0·993] | 471·7^**^  [173·0,770·4] | -0·144  [-0·373,0·0854] |
| 2^nd^ post-integration period | 0·944^*^  [0·902,0·987] | 130·4  [-121·0,381·8] | -0·561^***^  [-0·757,-0·364] |
| 3^rd^ post-integration period | 0·898^***^  [0·853,0·945] | 664·4^***^  [375·1,953·7] | -0·157  [-0·378,0·0638] |
| 4^th^ post-integration period | 0·938^**^  [0·895,0·983] | 254·9  [-4·165,514·0] | -0·478^***^  [-0·677,-0·280] |
| Luohu HMO Indicator | 0·889  [0·744,1·063] | -4879·0^***^  [-5259·4,-4498·6] | 0·206  [-0·182,0·594] |
| Age (years) | 1·008^***^  [1·006,1·010] | 105·9^***^  [94·05,117·8] | 0·0459^***^  [0·0368,0·0550] |
| Male | 1·116^***^  [1·080,1·154] | 1806·8^***^  [1614·5,1999·1] | 0·531^***^  [0·383,0·680] |
| Any hospitalization in the 90-day pre-index period | 1·676^***^  [1·603,1·752] | 1977·7^***^  [1695·4,2260·1] | 0·0157  [-0·198,0·229] |
| Days of inpatient stays in the 90-day pre-index period | 1·020^***^  [1·018,1·021] | -75·58^***^  [-88·43,-62·73] | 0·134^***^  [0·124,0·143] |
| Inpatient costs in the 90-day pre-index period (1,000 CN¥) | 1·020^***^ [1·001,1·003] | 99·00^***^ [91·77,106·2] | -0·0128^***^ [-0·0172,-0·00843] |
| Number of outpatient visits in the 90-day pre-index period | 1·016^***^  [1·012,1·020] | -142·5^***^  [-171·5,-113·5] | -0·0750^***^  [-0·0988,-0·0513] |
| Outpatient costs in the 90-day pre-index period (1,000 CN¥) | 0·999 [0·996,1·002] | 172·5^***^ [145·0,200·0] | 0·0467^**^ [0·0174,0·0760] |
| Indicators of diagnoses during the index hospitalization |  |  |  |
| *Essential hypertension* | 1·075^***^  [1·037,1·114] | 21·66  [-180·5,223·8] | 0·321^***^  [0·162,0·480] |
| *Type 2 diabetes* | 1·042^*^  [1·001,1·084] | 1269·7^***^  [1043·6,1495·7] | 1·054^***^  [0·877,1·231] |
| *Chronic ischemic heart disease* | 0·992  [0·945,1·042] | 623·5^***^  [354·2,892·9] | -0·272^*^  [-0·480,-0·0631] |
| *Atherosclerosis* | 0·844^***^  [0·803,0·887] | -2034·6^***^  [-2302·0,-1767·3] | 0·0136  [-0·193,0·220] |
| *Disorders of lipoprotein metabolism and other lipidemias* | 0·884^***^  [0·841,0·930] | -2813·9^***^  [-3085·1,-2542·6] | -0·571^***^  [-0·778,-0·363] |
| *Other liver diseases*  *(non-alcoholic fatty liver, congestion of liver, infarction of liver, etc.)* | 0·748^***^  [0·708,0·791] | -2117·2^***^  [-2399·4,-1835·1] | -0·0313  [-0·250,0·188] |
| *Heart failure* | 1·069^*^  [1·009,1·133] | 5979·5^***^  [5628·7,6330·3] | 0·297^*^  [0·0461,0·547] |
| *Cerebral infarction* | 1·092^**^  [1·032,1·154] | 3175·4^***^  [2856·9,3493·8] | 2·525^***^  [2·282,2·769] |
| *Sequelae of cerebrovascular disease* | 1·247^***^  [1·183,1·315] | 4981·8^***^  [4642·6,5320·9] | 4·974^***^  [4·720,5·228] |
| *Other disorders of fluid, electrolyte and acid-base balance (hyperosmolality and hypernatremia, acidosis, alkalosis, etc.)* | 1·077^*^  [1·012,1·146] | 6456·5^***^  [6072·0,6841·1] | 3·126^***^  [2·848,3·404] |
| *Gastritis and duodenitis* | 0·771^***^  [0·723,0·822] | -3924·8^***^  [-4259·1,-3590·6] | -0·364^**^  [-0·620,-0·109] |
| *Other cerebrovascular diseases (other cerebrovascular diseases, other cerebrovascular diseases, etc.)* | 0·913^**^  [0·855,0·975] | -3393·8^***^  [-3749·2,-3038·5] | -0·602^***^  [-0·875,-0·330] |
| *Cholelithiasis* | 1·017  [0·954,1·084] | 1227·7^***^  [880·3,1575·2] | 0·914^***^  [0·642,1·187] |
| *Other disorders of kidney and ureter (ischemia and infarction of kidney, cyst of kidney, etc.)* | 1·016  [0·956,1·080] | -171·0  [-501·4,159·4] | 0·632^***^  [0·372,0·892] |
| *Encounter for other aftercare and medical care* | 5·159^***^  [4·907,5·423] | -4236·4^***^  [-4628·6,-3844·1] | -3·656^***^  [-3·952,-3·361] |
| District indicators |  |  |  |
| *Guangming* | 1·265^***^  [1·183,1·352] | -5625·9^***^  [-6015·9,-5236·0] | 0·879^***^  [0·588,1·169] |
| *Nanshan* | 1·921^***^  [1·679,2·199] | -11960·3^***^  [-12853·4,-11067·1] | 2·276^***^  [1·608,2·943] |
| *Pingshan* | 1·243^***^  [1·186,1·301] | -3930·5^***^  [-4209·4,-3651·6] | -1·674^***^  [-1·883,-1·465] |
| *Dapeng* | 1·339^***^  [1·190,1·506] | -9782·5^***^  [-10499·7,-9065·4] | -0·281  [-0·823,0·260] |
| *Bao’an* | 1·068  [0·994,1·147] | -4866·3^***^  [-5267·3,-4465·3] | 2·038^***^  [1·737,2·340] |
| *Yantian* | 1·079  [0·951,1·224] | -7474·3^***^  [-8176·7,-6771·9] | -0·212  [-0·748,0·325] |
| *Futian* | 0·959  [0·890,1·033] | -6369·7^***^  [-6786·9,-5952·5] | -0·197  [-0·507,0·113] |
| *Longhua* | 1·348^***^  [1·174,1·549] | -12108·4^***^  [-12948·2,-11268·5] | -2·098^***^  [-2·729,-1·468] |
| *Longgang* | 1·107  [0·963,1·273] | -7631·5^***^  [-8449·8,-6813·3] | 0·982^**^  [0·354,1·609] |
| *N* | 120801 | 120801 | 120801 |

95% confidence intervals in brackets

^*^ *p* < 0·05, ^**^ *p* < 0·01, ^***^ *p* < 0·001

Abbreviations: OR, odds ratio; CI, 95% confidence interval; HMO, health maintenance organization; ROR, ratio of odds ratio.

Table S4. Results of the OLS regression of 90-day post-index outpatient costs using the Diff-in-diff specification

|  | 30-day readmission |
| --- | --- |
| Luohu HMO х1^st^ post-integration period | -66·34  [-200·7,68·00] |
| Luohu HMO х2^nd^ post-integration period | -120·3  [-249·1,8·408] |
| Luohu HMO х3^rd^ post-integration period | -43·33  [-172·0,85·33] |
| Luohu HMO х4^th^ post-integration period | -145·5^*^  [-284·2,-6·734] |
| 1^st^ post-integration period | 97·46^***^  [54·36,140·6] |
| 2^nd^ post-integration period | 160·6^***^  [112·1,209·2] |
| 3^rd^ post-integration period | 45·96  [-4·831,96·75] |
| 4^th^ post-integration period | 68·23^*^  [16·05,120·4] |
| Luohu HMO Indicator | -28·80  [-124·7,67·11] |
| Age (years) | -6·152^***^  [-8·228,-4·077] |
| Male | 50·76^***^  [20·68,80·84] |
| Any hospitalization in the 90-day pre-index period | 50·10  [-6·619,106·8] |
| Days of inpatient stays in the 90-day pre-index period | -1·786  [-4·402,0·829] |
| Inpatient costs in the 90-day pre-index period (1,000 CN¥) | 1·162  [-1·377,3·701] |
| Number of outpatient visits in the 90-day pre-index period | -24·70  [-140·2,90·76] |
| Outpatient costs in the 90-day pre-index period (1,000 CN¥) | 728·5^***^  [509·1,948·0] |
| Indicators of diagnoses during the index hospitalization |  |
| *Essential hypertension* | 79·48^***^  [47·02,112·0] |
| *Type 2 diabetes* | 125·0^***^  [73·43,176·6] |
| *Chronic ischemic heart disease* | 23·36  [-37·33,84·05] |
| *Atherosclerosis* | 80·76^***^  [43·97,117·6] |
| *Disorders of lipoprotein metabolism and other lipidemias* | 77·01^**^  [29·38,124·6] |
| *Other liver diseases*  *(non-alcoholic fatty liver, congestion of liver, infarction of liver, etc.)* | -37·17  [-74·38,0·0451] |
| *Heart failure* | 275·2^***^  [192·3,358·0] |
| *Cerebral infarction* | -105·7^***^  [-157·5,-53·86] |
| *Sequelae of cerebrovascular disease* | -110·1^***^  [-156·4,-63·77] |
| *Other disorders of fluid, electrolyte and acid-base balance (hyperosmolality and hypernatremia, acidosis, alkalosis, etc.)* | -54·99  [-133·2,23·25] |
| *Gastritis and duodenitis* | -80·58^***^  [-124·3,-36·86] |
| *Other cerebrovascular diseases (other cerebrovascular diseases, other cerebrovascular diseases, etc.)* | 80·10^*^  [7·603,152·6] |
| *Cholelithiasis* | 57·90  [-19·13,134·9] |
| *Other disorders of kidney and ureter (ischemia and infarction of kidney, cyst of kidney, etc.)* | 36·40  [-12·94,85·74] |
| *Encounter for other aftercare and medical care* | 332·3^***^  [226·1,438·4] |
| District indicators |  |
| *Guangming* | -101·3^*^  [-185·1,-17·47] |
| *Nanshan* | -156·3  [-321·2,8·632] |
| *Pingshan* | 66·92^**^  [16·69,117·1] |
| *Dapeng* | -40·43  [-256·1,175·3] |
| *Bao’an* | 151·9^***^  [66·40,237·4] |
| *Yantian* | -12·57  [-108·6,83·44] |
| *Futian* | -16·74  [-118·6,85·13] |
| *Longhua* | -71·14  [-200·6,58·32] |
| *Longgang* | -172·9^***^  [-262·1,-83·64] |
| *N* | 120801 |

Standard errors in parentheses

^*^ *p* < 0·05, ^**^ *p* < 0·01, ^***^ *p* < 0·001

Abbreviations: OLS, ordinary least squares; HMO, health maintenance organization.

Table S5. Results of the tests of parallel trends across groups using multivariate OLS regressions by including Luohu model indicator by month interaction terms

|  | 30-day readmission | Costs of hospitalization episode | Length of stay |
| --- | --- | --- | --- |
| April | 0·00804  (0·00679) | 1550·0^**^  (530·7) | 0·0317  (0·254) |
| May | 0·0148^*^  (0·00683) | 1073·5^*^  (522·3) | 0·216  (0·275) |
| June | 0·00353  (0·00677) | 3230·3^***^  (751·6) | 1·403^***^  (0·368) |
| July |  | Reference month |  |
| Luohu HMO | -0·0366^*^  (0·0174) | -2877·1  (1920·3) | -0·461  (0·694) |
| Luohu HMO х April | 0·0367  (0·0264) | -3754·3  (2267·8) | 0·397  (0·877) |
| Luohu HMO х May | 0·0336  (0·0273) | -1673·9  (2469·0) | -0·175  (0·985) |
| Luohu HMO х June | 0·00722  (0·0244) | -1822·2  (3240·1) | 2·791  (2·280) |
| Luohu HMO х July |  | Reference month |  |
| *N* | 22820 | 22820 | 22820 |

Standard errors in parentheses

^*^ *p* < 0·05, ^**^ *p* < 0·01, ^***^ *p* < 0·001

Results are presented as coefficients (standard errors) unless otherwise specified.


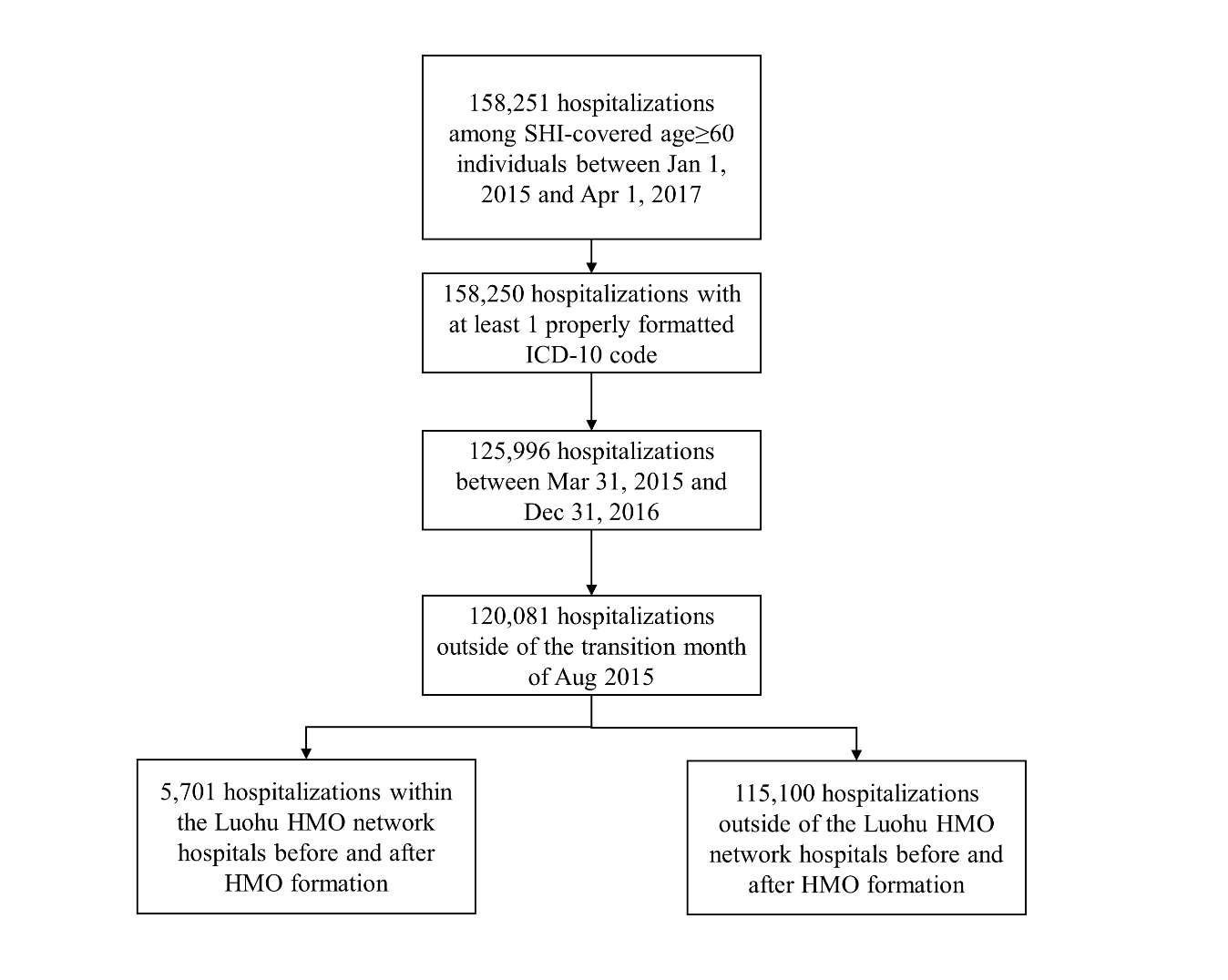


Fig S1. The flowchart of sample selection.


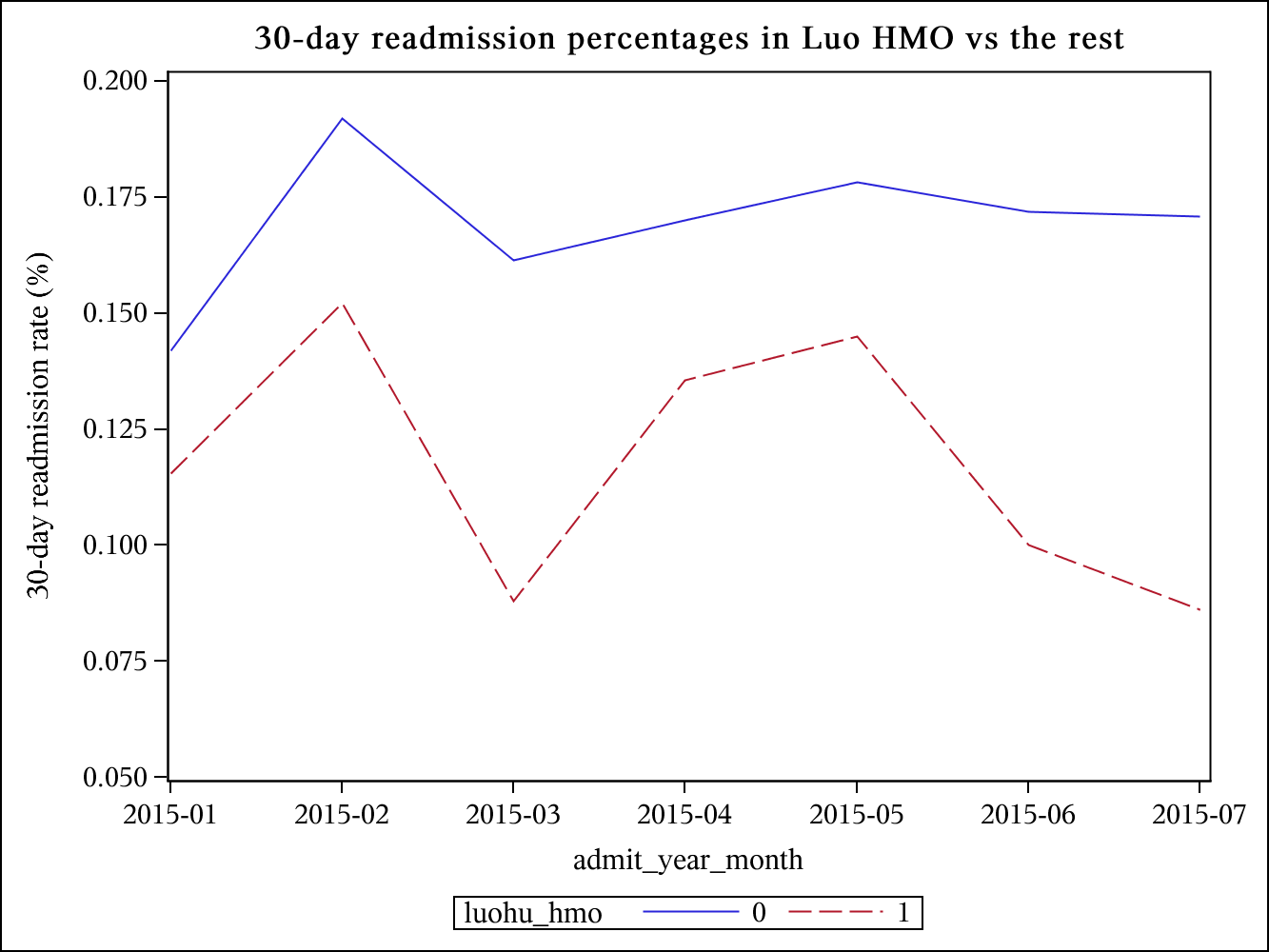


Fig S2. The pre-index trends of 30-day readmission rates in the intervention and counterfactual groups.


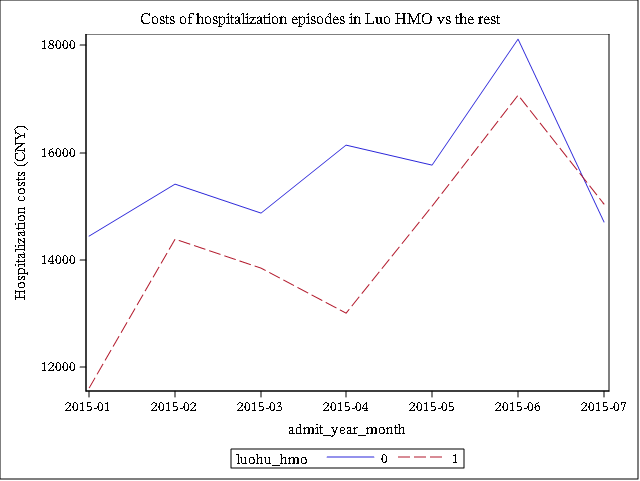


Fig S3. The pre-index trends of inpatient costs among the intervention and counterfactual groups.


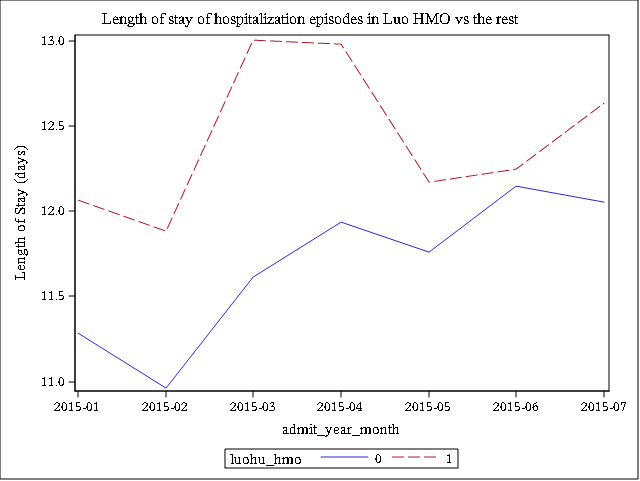


Fig S4. The pre-index trends of length of stays among the intervention and counterfactual groups.
